# West Midlands Oncology Association trials of adjuvant chemotherapy in operable breast cancer: results after a median follow-up of 7 years. II. Patients without involved axillary lymph nodes.

**DOI:** 10.1038/bjc.1989.390

**Published:** 1989-12

**Authors:** J. M. Morrison, A. Howell, K. A. Kelly, R. J. Grieve, I. J. Monypenny, R. A. Walker, J. A. Waterhouse

**Affiliations:** Selly Oak Hospital, Birmingham, UK.

## Abstract

The aim of this study was to test the effectiveness of a regimen of combination chemotherapy when given as an adjuvant treatment after mastectomy to patients with histologically negative axillary lymph nodes. A total of 574 patients with cancer of the breast and no involvement of axillary lymph nodes were randomised, after simple mastectomy with axillary sampling, to receive either no adjuvant treatment or oral fluorouracil 500 mg, methotrexate 25 mg and chlorambucil 10 mg p.o. on day 1 and fluorouracil 500 mg and chlorambucil 10 mg p.o. on day 2 (LMF) every 21 days for eight cycles. Randomisation was stratified according to menopausal status and tumour size. Treatment was started within 14 days of surgery in 97% of patients. Ninety per cent of patients received eight cycles of chemotherapy with no dose reduction. At a median follow-up of 7 years, there was no evidence that relapse-free or overall survival time were influenced by treatment.


*Br. J. Cancer* (1989), **60**, 919–924 © The Macmillan Press Ltd., 1989

### West Midlands Oncology Association trials of adjuvant chemotherapy in operable breast cancer: results after a median follow-up of 7 years. II Patients without involved axillary lymph nodes

J.M. Morrison[1], A. Howell[2], K.A. Kelly[3], R.J. Grieve[4], I.J. Monypenny[5], R.A. Walker[6] & J.A.H. Waterhouse[7]

[1]*Selly Oak Hospital, Birmingham, UK;* [2]*Christie Hospital, Manchester, UK;* [3]*West Midlands Cancer Research Campaign Clinical Trials Unit, Birmingham, UK;* [4]*Walsgrave Hospital, Coventry, UK;* [5]*Llangdough Hospital, Cardiff, UK;* [6]*Department of Pathology, University of Leicester, UK; and* [7]*West Midlands Regional Cancer Registry, Birmingham, UK.*

**Summary** The aim of this study was to test the effectiveness of a regimen of combination chemotherapy when given as an adjuvant treatment after mastectomy to patients with histologically negative axillary lymph nodes. A total of 574 patients with cancer of the breast and no involvement of axillary lymph nodes were randomised, after simple mastectomy with axillary sampling, to receive either no adjuvant treatment or oral fluorouracil 500 mg, methotrexate 25 mg and chlorambucil 10 mg p.o. on day 1 and fluorouracil 500 mg and chlorambucil 10 mg p.o. on day 2 (LMF) every 21 days for eight cycles. Randomisation was stratified according to menopausal status and tumour size. Treatment was started within 14 days of surgery in 97% of patients. Ninety per cent of patients received eight cycles of chemotherapy with no dose reduction. At a median follow-up of 7 years, there was no evidence that relapse-free or overall survival time were influenced by treatment.

Although patients with node negative breast cancer are thought to have a good prognosis, approximately 25% will have relapsed 5 years after primary treatment (Friedman *et al.*, 1986). It is clear from extensive clinical experience that relapse is almost always associated with subsequent death from breast cancer. Thus, if approximately one-quarter of patients with node negative breast cancer die of their disease, this is only a good prognosis in relation to node positive disease; it is a poor prognosis in relation to no disease.

Patients with node negative disease who relapse do so on average later than those with node positive disease (Nissen-Meyer *et al.*, 1986). This suggests that at presentation they have a smaller tumour burden. Since experimental data indicate that adjuvant chemotherapy is more active the smaller the tumour burden, these patients should theoretically benefit from treatment more than those with node positive disease.

We therefore decided to test the hypothesis that patients with node negative disease would benefit from chemotherapy by using oral chlorambucil, methotrexate and fluorouracil (LMF). Treatment was to begin as soon as possible after mastectomy in view of the Nissen-Meyer study which showed an advantage for early treatment (Nissen-Meyer *et al.*, 1986).

The trial was started in December 1976 as a multicentre study within the West Midlands region of the United Kingdom. Preliminary analyses of the study were reported when the median follow-up times were 22, 54 and 60 months (Morrison *et al.*, 1981, 1984, 1987). This paper presents a more complete analysis of the trial 10 years after recruitment began when the median follow-up was 7 years.

### Patients and methods

### *Selection of patients*

Patients were entered into the trial by 40 consultant surgeons from 26 hospitals within the West Midlands Region between December 1976 and August 1984. Patients without axillary lymph node involvement, $T_{1a}$–$T_{3a}$ tumours and below the age of 65 were eligible. Additional eligibility criteria were: WBC $>4.0 \times 10^9\,l^{-1}$, platelet count $>100 \times 10^9\,l^{-1}$, normal liver function tests, not pregnant or lactating, no previous malignancy except rodent ulcer, squamous cell carcinoma of skin or carcinoma *in situ* of cervix, no serious intercurrent disease or psychiatric disorder and ability to be followed up adequately. Mandatory initial investigations were full blood count, biochemical profile, liver function tests, ECG, chest and skeletal radiographs and a radioisotope bone scan.

### *Treatment*

Following histological confirmation of the diagnosis all patients had a simple mastectomy with axillary node sampling (Forrest *et al.*, 1976). Postoperative radiotherapy was not given. After confirmation of axillary node status, patients were randomised by telephone call to the West Midlands Cancer Registry and after September 1983 to the West Midlands Cancer Research Campaign Clinical Trials Unit to receive either no further treatment or chemotherapy. Prospective stratification was made for menopausal status and tumour size ($<5$ cm; $\geqslant 5$ cm). Chemotherapy was started within 7 days of surgery in 81% of patients and 14 days in 97%. A further seven cycles were given every 21 days on an outpatient basis. Patients received orally fluorouracil 500 mg, methotrexate 25 mg and chlorambucil 10 mg on day 1. On day 2 they received orally fluorouracil 500 mg and chlorambucil 10 mg. Courses were delayed if there was evidence of significant myelosuppression (WBC $<3 \times 10^9\,l^{-1}$; platelets $<100 \times 10^9\,l^{-1}$) or other serious toxic manifestations. There were no dosage reductions. Treatment after relapse was decided by clinicians individually.

### *Assessment*

Patients were examined every 3 months for 18 months and thereafter at 6-monthly intervals until recurrence or death. A full blood count, biochemical profile and liver function tests were performed at each visit. Chest and skeletal X-rays and bone scans were performed 6-monthly for 2 years followed by annual investigations until 5 years. Toxicity was recorded for each treatment cycle. Nausea and vomiting were graded as mild or severe on a subjective basis by each surgical team. Clinicians were also asked to report the duration of major symptoms in days.

### *Pathology and receptors*

Histology was reviewed centrally by one of us (R.A.W.) and classified according to histological type and grade; the

Correspondence: J.M. Morrison.
Received 6 March 1989; and in revised form 31 July 1989.

grading used (Elston et al., 1982) was a modification of the system used by Bloom and Richardson (1957). Oestrogen and progesterone receptors were assayed using the dextran coated charcoal method and Scatchard analysis (McGuire & De La Garza, 1973). Receptors were taken to be present if ⩾5 fmol per mg cytosol protein were detectable.

*Audit*

All recurrences were verified histologically if superficial, or by radiology and scanning if not, and reviewed by one of us (J.M.M.). External audit was performed by Dr T.J. Powles in November 1985. Computer files, trial forms, clinical notes and X-rays were examined for every twentieth patient and for a random sample of patients with recurrence in the bone and lung.

*Statistical analyses*

The major end-points of the trial were histologically or radiologically defined recurrence and death. The completeness of the notification of death was verified by registration of all patients with the West Midlands Regional Cancer Registry, Birmingham, and the National Health Service Central Register, Southport. In accordance with accepted statistical practice, this permitted all randomised patients to be included in the survival analysis (Peto et al., 1977). However, since there was no notification by clinicians of disease status for patients who were randomised but were found subsequently to be ineligible, only eligible patients are included in the analysis of recurrence, relapse-free survival and toxicity. Relapse-free survival and overall survival curves were drawn using the method of Kaplan and Meier (1958) and the significance of the differences between curves assessed using the log rank test (Peto et al., 1977). Treatment comparisons were stratified by menopausal status and tumour size. In addition the effect of controlling for menopausal status, tumour size, age, tumour grade and receptor content was determined using Cox's multiple regression analysis (Cox, 1972). The reduction in the odds of relapse and death (Early Breast Cancer Trialists' Collaborative Group, 1988) and relative improvements were calculated. Patients with uncertain menopausal status (e.g. previous hysterectomy) were taken to be premenopausal if less than 50 years and post-menopausal if 50 years or more. Patients were taken to be post-menopausal if they had had no periods within the previous 6 months.

### Results

*Patients analysed*

A total of 574 patients were randomised (285 treated and 289 control) of whom 543 (273 treated and 270 control) were eligible according to the criteria given above. Data were censored at 31 December 1986 when the median follow-up was 7 years. Analysis of survival includes all randomised patients whereas other analyses were performed on eligible patients only; the 31 ineligible patients (13 treated and 18 control) were not followed up. Patients were excluded after randomisation for the following reasons: nodes not sampled or node positive (four treated, five control); advanced disease (two treated); too old (two treated, five control); white blood count too low (one control); abnormal LFTs (two treated, four control); other malignancy (one treated, two control); carcinoma *in situ* (one treated); benign lump (one control); intercurrent disease (one treated). One treated patient was completely lost to follow-up and is not included in the analysis of recurrence. The characteristics of the eligible patients are given in Table I, which shows that there are no major imbalances of prognostic factors between the treated and control groups.

*Relapse-free survival*

Adjuvant chemotherapy had no significant effect on relapse-free survival (Figure 1, Table II). The percentage relapse-free at 5 years is 73% (95% confidence interval (CI) 67–78%) in the treated and 71% (CI 65–76%) in the control group, which represents a relative improvement (RI) of 3% in the relapse rate at 5 years or an 11% reduction in the odds of relapse (OR). After stratification for menopausal status and tumour size, the $\chi^2_1$ is 0.51 ($P = 0.47$). Controlling for menopausal status, tumour size, age, grade and receptor status does not alter the result. For comparability with other

**Table I** Patient characteristics

| | *Treated (273 patients)* | | *Control (270 patients)* | |
|---|---|---|---|---|
| | *n* | % | *n* | % |
| Age | | | | |
| Less than 50 | 119 | 44 | 127 | 47 |
| 50 plus | 154 | 56 | 143 | 53 |
| Menopausal status | | | | |
| Premenopausal | 129 | 48 | 128 | 47 |
| Post-menopausal | 116 | 42 | 120 | 45 |
| Hysterectomy | 28 | 10 | 22 | 8 |
| Tumour size | | | | |
| <2.0 | 51 | 19 | 64 | 24 |
| 2.0–4.9 | 184 | 67 | 180 | 67 |
| ⩾5.0 | 38 | 14 | 26 | 9 |
| Histology | | | | |
| Infiltrating duct | 199 | 73 | 194 | 72 |
| Infiltrating lobular | 27 | 10 | 38 | 14 |
| Other | 27 | 10 | 22 | 8 |
| Not reviewed | 20 | 7 | 16 | 6 |
| Grade | | | | |
| I | 38 | 14 | 44 | 16 |
| II | 144 | 53 | 153 | 57 |
| III | 71 | 26 | 54 | 20 |
| Not reviewed | 20 | 7 | 19 | 7 |
| Oestrogen receptors | | | | |
| Negative | 61 | 22 | 51 | 19 |
| Positive | 121 | 44 | 124 | 46 |
| Not assayed | 91 | 33 | 95 | 35 |
| Progresterone receptors | | | | |
| Negative | 67 | 25 | 75 | 28 |
| Positive | 82 | 30 | 74 | 27 |
| Not assayed | 124 | 45 | 121 | 45 |

**Figure 1** Relapse-free interval for all eligible patients. LMF (81/272 relapsed); control (88/270 relapsed); $\chi^2_1 = 0.58$; $P = 0.45$.

studies, the results are broken down by menopausal status and age in Table II. Since there was no effect of chemotherapy overall, further subgroup analyses are not presented.

There were 81 (30%) patients who recurred in the treated group and 88 (33%) in the control group. Although the total number of recurrences is similar in both groups, there was a highly significant difference in the distribution of metastases between the treated and control groups ($\chi^2_1 = 8.5$; $P = 0.0004$), 65% having distant recurrence in the treated group and only 43% in the control group (Table III). An equal number of local recurrences is expected in the treated and control groups, but there were significantly more local recurrences in the control group than expected on the basis of a 1:1 ratio ($\chi^2_1 = 6.5$; $0.02 > P > 0.01$). However, although there was a greater number of distant recurrences than expected in the treated group, the deviation from a 1:1 ratio was not significant ($\chi^2_1 = 2.4$; $0.2 > P > 0.1$).

**Table III** Sites of first recurrence

| | *Treated (81)* *n (%[a])* | *Control (88)* *n (%[a])* |
|---|---|---|
| Local and regional recurrence only | 28 (35) | 50 (57) |
| Total with distant recurrence | 52 (65) | 37 (43) |
| Breakdown of distant recurrences | | |
| Bone | 20 (25) | 23 (26) |
| Liver | 2 (2) | 1 (1) |
| Lung | 15 (19) | 5 (6) |
| Other[b] | 12 (15) | 4 (5) |
| Distant and local recurrence | 3 (4) | 4 (5) |
| Not reviewed[c] | 1 | 1 |

[a]% of those reviewed. [b]Ascites (3T), brain (1T,1C), contralateral breast (8T,1C), contralateral axilla (2C). [c]Died with disease, but no recurrence recorded.

**Figure 2** Survival for all randomised patients. LMF (56/285 died); control (56/289 died); $\chi^2_1 = 0.02$; $P = 0.88$.

### *Survival*

There was no significant effect of chemotherapy on overall survival (Figure 2). After stratification for menopausal status and tumour size, $\chi^2_1 = 0.11$; $P = 0.74$. Controlling for menopausal status, tumour size, age, tumour grade and receptor status does not alter this result. The results overall and broken down by menopausal status and tumour size are shown in Table II.

### *Chemotherapy*

Ninety percent of patients received eight cycles of treatment, 93% seven cycles or more and 96% four cycles or more; all patients received at least two cycles of chemotherapy. The reasons for not receiving the full course of eight cycles of chemotherapy were toxicity (15%), intercurrent illness not associated with treatment (3%), patient refusal (30%) and administrative errors mainly involving failure to give the eighth cycle (52%).

### *Toxicity*

The proportion of patients affected and the number of cycles in which toxic effects were seen in summarised in Table IV. Severe leukopenia and thrombocytopenia were rare. Most patients had nausea and vomiting on at least one occasion. When these symptoms were analysed on a per cycle basis, 69% of cycles were associated with nausea and 32% with vomiting. Treatment was delayed by one week in 74 cycles and longer in 39. Mild alopecia occurred in a third of patients, while other side-effects such as stomatitis or neuropathy were uncommon.

The severity and duration of nausea and vomiting are outlined in Figure 3. Severe symptoms were uncommon. However, when symptoms occurred, they lasted for longer than 24 hours in approximately a third of the cycles assessed.

An assessment of the 'quality of life' of patients is shown in Figure 3. Patients were asked how long they were unwell, how long they were unable to go to work (or perform housework) and how long they were confined to bed if they

**Table II** Effect of treatment in subgroups of patients

| | *Relapse-free survival* | | | | | | | | *Overall survival* | | | | | | | |
|---|---|---|---|---|---|---|---|---|---|---|---|---|---|---|---|---|
| | *Treated* | | *Control* | | | | | | *Treated* | | *Control* | | | | | |
| | *N* | *R* | *N* | *R* | $\chi^2_1$ | *P* | *RI* | *OR* | *N* | *D* | *N* | *D* | $\chi^2_1$ | *P* | *RI* | *OR* |
| Overall | 272 | 81 | 270 | 88 | 0.58 | 0.45 | 3 | 11 | 285 | 56 | 289 | 56 | 0.02 | 0.88 | 0 | −3 |
| Menopausal status | | | | | | | | | | | | | | | | |
| Pre | 137 | 38 | 134 | 47 | 1.51 | 0.22 | 6 | 23 | 143 | 27 | 139 | 28 | 0.07 | 0.79 | −3 | 7 |
| Post | 135 | 43 | 136 | 41 | 0.02 | 0.89 | 0 | −3 | 140 | 29 | 150 | 28 | 0.23 | 0.63 | 1 | −14 |
| Age | | | | | | | | | | | | | | | | |
| <50 | 118 | 36 | 127 | 42 | 0.19 | 0.67 | 2 | 9 | 122 | 25 | 131 | 27 | 0.00 | 0.96 | −2 | 1 |
| ⩾50 | 154 | 45 | 143 | 46 | 0.36 | 0.55 | 4 | 12 | 163 | 31 | 158 | 29 | 0.07 | 0.79 | 1 | −7 |

N, number of patients in subgroup; R, number of patients who have relapsed; D, number of patients who have died; RI, relative improvement; OR, odds reduction.

**Table IV** Toxicity attributed to chemotherapy

| | *Patients* | | *Cycles* | |
|---|---|---|---|---|
| *Haematology* | *n (259)* | *%* | *n (1746)* | *%* |
| Hb (g $dl^{-1}$) | | | | |
| 9.5–10.9 | 26 | 10 | 57 | 5 |
| <9.5 | 4 | 2 | 4 | <1 |
| WBC count ($\times 10^9 l^{-1}$) | | | | |
| 3–3.9 | 88 | 34 | 280 | 30 |
| 2–2.9 | 5 | 2 | 5 | <1 |
| <2 | 0 | 0 | 1 | <1 |
| Platelets ($\times 10^9 l^{-1}$) | | | | |
| 70–99 | 4 | 2 | 1 | ~0 |
| <70 | 2 | 1 | 2 | ~0 |
| *Side-effects* | *n (259)* | *%* | *n (1877)* | *%* |
| Nausea | 243 | 94 | 1300 | 69 |
| Vomiting | 184 | 71 | 606 | 32 |
| Rash | 40 | 15 | 55 | 3 |
| Stomatitis | 62 | 24 | 110 | 6 |
| Diarrhoea | 66 | 25 | 133 | 7 |
| Neurological | 62 | 24 | 112 | 6 |
| Mild hair loss | 88 | 34 | | |
| Cardiac failure | 0 | 0 | | |

had toxicity. Patients were unwell in 57% of cycles. In 30% of cycles patients took time off work and in 19% they were confined to bed for a variable period.

*Second primary tumours*

Second primary tumours occurred in three of the controls (colon, multiple myeloma, ovary) and three of the treated group (colon, lung, pancreas). No leukaemias have been recorded.

### Discussion

After a median follow-up of 7 years, patients treated with oral LMF did not have a relapse-free or overall survival advantage compared with untreated controls. Although the regimen of chemotherapy was designed to be non-toxic, it proved to have much more toxicity than anticipated from pilot studies. Indeed, the toxicity of this regimen appears similar to the intravenous chemotherapy described in the previous paper, with the exception of alopecia. It may be more myelotoxic than AVCMF in susceptible patients since the WBC immediately before the subsequent course was between 3.0 and $3.9 \times 10^9 l^{-1}$ in 30% of LMF cycles and 14% of only AVCMF cycles. However, in spite of showing activity in terms of myelosuppression, there was no detectable anti-tumour effect in terms of relapse-free or overall survival.

The sites of first recurrence in this study were paradoxical. Although the total number of recurrences was very similar, there were significantly fewer local and regional recurrences in treated patients (28 in the treated group and 50 in the controls; $\chi^2_1 = 6.5$; $0.2 > P > 0.01$) whereas there were rather more distant recurrences in treated patients (52 *vs* 37; $\chi^2_1 = 2.4$; $0.2 > P > 0.1$), with the excess attributable to recurrences in lung, contralateral breast and ascites. Explanations for this effect must be conjectural and include chance, immunosuppression or chemotherapy induced sublethal cell damage leading to genetic alteration and subsequent increased growth rates. This paradoxical phenomenon has not led to a significantly worse survival in the treated arm.

The results of other trials comparing the use of prolonged combination chemotherapy with a no treatment control arm in node negative patients are shown in Table V (Koyama *et al.*, 1980; Senn *et al.*, 1986; Semiglazov *et al.*, 1986; Jakesz *et al.*, 1987; Ludwig Breast Cancer Study Group, 1989; Espie *et al.*, 1987; Bonadonna *et al.*, 1987; Williams *et al.*, 1987; Mansour *et al.*, 1989; Fisher *et al.*, 1989). In most, low dose oral or intravenous treatments have been studied in node

**Figure 3** Side-effects of treatment: nausea and vomiting and aspects of quality of life.

**Table V** Randomised trials of adjuvant polychemotherapy compared with untreated controls in node negative patients

| *Author (study)* | *Period of accrual*[a] | *n*[b] | *Regimen*[c] | *Effect on relapse* *P value*[d] | *% RF*[e] | *Effect on survival* *P value*[d] | *% alive*[e] | *Median follow-up* | *Date of report* |
|---|---|---|---|---|---|---|---|---|---|
| *Trials with unselected node negative patients* | | | | | | | | | |
| Koyama (Japanese) | 62–76 | 229 | C and/or Mmc | NS | 94/90 (5) | NS | – | ~9 years | 80 |
| Senn (OSAKO) | 74–77 | 122 | LMF | S | – | NS | – | 9 years | 86 |
| Semiglazov (USSR) | 75–82 | 252 | CMF/TMF | <0.05 | 90/81 (5) | <0.10 | 87/80 (5) | ~6 years | 86 |
| Morrison (WMOA) | 76–84 | 574 | LMF | 0.45 | 77/74 (4) | 0.88 | 91/90 (4) | 7 years | 89 |
| Jakesz (Viennese) | 77–81 | 128 | CMFVb | – | – | <0.02 | 90/76 (0) | 6 years | 87 |
| Ludwig (Swiss) | 81–85 | 1275 | CMF | 0.04 | 77/73 (4) | 0.24 | 90/86 (4) | 3.5 years | 89 |
| Espie (Paris) | – | 165 | CMF | NS | 59/53 (6) | NS | 82/92 (6) | 3 years | 87 |
| *Trials with ER negative node negative patients* | | | | | | | | | |
| Bonadonna (Milan) | 80–85 | 90 | CMF | <0.001 | 89/53 (4) | 0.02 | 93/63 (4) | ~3 years | 87 |
| Williams (Southampton) | 81–85 | 52 | VAP | 0.001 | 95/68 (O) | – | 95/87 (0) | 3 years | 87 |
| Mansour (ECOG) | 81–88 | 406 | CMFP | 0.0001 | 84/69 (3) | NS | – | 3 years | 89 |
| Fisher (NSAPB) | 81– | 679 | MF | 0.003 | 80/71 (4) | 0.8 | 86/87 (4) | 4 years | 89 |

[a]Analysis may be based on a shorter period for which there is adequate follow-up. [b]Analysis based on this number of patients, more may have been randomised. [c]C, cyclophosphamide; Mmc, mitomycin C; L, chlorambucil; M, methotrexate; F, fluorouracil; T, thiotepa; Vb, vinblastine; A, adriamycin; P, prednisone. [d]NS, $P > 0.05$; S, $P \leqslant 0.05$; $P$ for log rank test except $t$ test for Semiglazov. [e]% in treated group/% in control group (year at which percentages given). O, over the observation period; Semiglazov gives distant relapses only.

negative patients irrespective of receptor status. With the exception of the Viennese study (Jakesz, 1987), there have been no reported improvements in survival in this type of study, which is not surprising in view of the small absolute improvement possible in this good prognosis group and the small sample sizes. Several studies report marginal improvements in relapse free survival (Senn *et al.*, 1986; Semiglazov *et al.*, 1986; Ludwig Breast Cancer Study Group, 1989). There is no significant improvement in either relapse-free or overall survival in our trial. This could be due to the chemotherapy used. In the overview of the Early Breast Cancer Trialists' Collaborative Group (1988) CMF-based regimens gave greater reductions in odds than regimens without all or some of C, M, F and single agents, although there was no significant heterogeneity between types of regimen. However, Senn *et al.* (1986) report significantly improved relapse-free survival with LMF and our failure to demonstrate any significant benefit could be due to chance and the relatively small number of patients studied for the magnitude of improvement that now seems likely in this group.

These trials in unselected node negative patients are confounded not only by the relatively small numbers of patients but also by the relatively low number and late occurrence of events in node negative patients. More recent trials have tried to select high risk groups on the basis of oestrogen receptor status. These studies have all demonstrated highly significant improvements in relapse free survival. In the NSAPB study (Fisher *et al.*, 1989), 741 node negative, ER negative patients were randomised to receive methotrexate $100\,mg\,m^{-2}$ i.v. days 1 and 8 every 4 weeks and fluorouracil $600\,mg\,m^{-2}$ i.v. days 1 and 8 one hour after methotrexate for a period of 12 months or no further treatment. At 4 years there was a 9% reduction in the absolute number of recurrences in the chemotherapy treated group (relapse-free survival 80% *vs* 71%). ECOG (Mansour *et al.*, 1989) randomised 536 node negative, ER negative patients to receive cyclophosphamide $100\,mg\,m^{-2}$ and prednisone $40\,mg\,m^{-2}$ orally days 1–14 with methotrexate $40\,mg\,m^{-2}$ i.v. and fluorouracil $600\,mg\,m^{-2}$ i.v. days 1 and 8, repeated 4-weekly. At 3 years there was a highly significant improvement in relapse-free survival (84% *vs* 69%). Bonadonna *et al.* (1987) randomised 90 node negative, ER negative patients to receive cyclophosphamide $600\,mg\,m^{-2}$, fluorouracil $600\,mg\,m^{-2}$ and methotrexate $40\,mg\,m^{-2}$ i.v. every 21 days for 12 cycles. At 3 years there was a significant improvement in relapse-free survival (89% *vs* 53%) and overall survival (93% *vs* 63%).

Although there was no heterogeneity between types of regimen in the overview, it may well be that less intensive regimens do not provide adequate treatment. If the NSAPB, ECOG and Milan results are meaningful, it might suggest that if node negative patients are treated with adjuvant chemotherapy, high risk groups should be selected and regimens shown to be active in node positive disease used in full doses.

Participating surgeons and hospitals: Birmingham General Hospital: J. Alexander-Williams, R.M. Baddeley, N.J., Dorricot, M.R.B. Keighley, G.D. Oates; Bromsgrove General Hospital: J.H. Burman, G.F. Grave; Burton District Hospital and Burton General Hospital: H.C. De Castella, S. Glick; Dudley Road Hospital, Birmingham: P.G. Bevan, I.A. Donovan, M. Obeid; Edgbaston Nursing Home, Birmingham; George Eliot Hospital, Nuneaton: J.R. Moffat; Good Hope District General Hospital, Sutton Coldfield: W.M. Lien, R.S. Rihan, D.R. Thomas; Kidderminster General Hospital: P.R. Armistead, R.E. Gibbons, E.W. Gillison; Longton Cottage Hospital; North Staffordshire Royal Infirmary, Stoke-on-Trent: L.J. Lawson, E.R. Monypenny; Queen Elizabeth Hospital, Birmingham: J. Fielding, J.D. Hamer, J.G. Temple; Sandwell District General Hospital, West Bromwich: J.D. Hennessy; Selly Oak Hospital, Birmingham: J.P. Grant, A.R. Leask, J. Morrison, N.E. Winstone; Solihull Hospital: R.W. Tudor; St Chad's Hospital, Birmingham of St Cross, Rugby: T.A. Waterworth; Stratford-upon-Avon Hospital: R.T. Marcus; Victoria Hospital, Lichfield: F.R. Hurford; Walsall General Hospital and Manor Hospital, Walsall: K.D. Fortes-Mayer; Walsgrave Hospital: G.A. Court, R.W. Parker; Warneford General Hospital, Leamington Spa: M.D. Lord; Warwick General Hospial: J.D. Marsh; Worcester Royal Infirmary: H.T. Williams; Wordsley Hospital, Stourbridge: H. Kramer.

We wish to thank the Cancer Research Campaign for grants to the project, Eli Lilly, Farmitalia Carlo Erba and Lederle for their financial assistance and also the Lions Club International District 105 and the many other groups and individuals within the West Midlands who provided support by fund raising activities or donation. In particular, we thank Lady Veronica Booth for working so hard as patron of our local fund raising campaign. Members of the WMOA Breast Cancer Study Group were K. Arthur, A. Banks, W. Bond, D. Cove, I. Donovan, C. Fortes-Meyer, J. Harnden, A. Howell, E.R. Monypenny, J.M. Morrison, C. Newman, G.D. Oates, A. Rowse, R. Walker, J.A.H. Waterhouse and K. Woods. Mr Alan Hughes and Miss Sharon Hughes carried out the receptor estimation. We are most grateful to Miss Sally Burman, Mrs Jane Gaines, Mr Alan Marson, Dr Abe Minawa, Mrs Linda Pitt, Mrs Linda Ward and Ms Wendy Gillespie for their help in preparing the data. We thank all the staff of the West Midlands Regional Cancer Registry and West Midlands Cancer Research Campaign Clinical Trials Unit who have given assistance. The co-operation of participating surgeons, pathologists, radiologists and medical physicists throughout the West Midlands has been crucial to the success of the trial and we wish to acknowledge their continuing support. We also wish to thank Dr T. Powles for his hard work in carrying out the trial audit.

### References

BLOOM, H.J.G. & RICHARDSON, W.W. (1957). Histological grading and prognosis in breast cancer. *Br. J. Cancer*, **11**, 359.

BONADONNA, G., VALAGUSSA, T., ZAMBETTI, M., BUZZONI, R. & MOLITERNI, A. (1987). Milan adjuvant trials for Stage I-II breast cancer. In *Adjuvant Therapy of Cancer V*, Salmon, S.E. (ed.) p. 211. Grune & Stratton: Orlando, FL.

COX, D.R. (1972). Regression models and life tables. *J. R. Stat. Soc. Series B*, **34**, 187.

EARLY BREAST CANCER TRIALISTS' COLLABORATIVE GROUP. (1988). Effects of adjuvant tamoxifen and of cytotoxic therapy on mortality in early breast cancer. *N. Engl. J. Med.*, **319**, 1681.

ELSTON, C.W., GRESHAM, T.A., RAO, G.S. *et al.* (1982). The Cancer Research Campaign (King's/Cambridge) trial for early breast cancer: clinico-pathological aspects. *Br. J. Cancer*, **45**, 655.

ESPIE, M., COLIN, P., MIGNOT, L. & 6 others (1987). Adjuvant chemotherapy in premenipausal women with node-negative breast adenocarcinoma: interim analysis of a randomized trial. In *ECCO-4. Fourth European Conference on Clinical Oncology and Cancer Nursing*, p. 114.

FISHER, B., REDMOND, C., DIMITROV, N.V. & 10 others (1989). A randomized clinical trial evaluating sequential methotrexate and flourouracil in the treatment of patients with node-negative breast cancer who have estrogen-receptor-negative tumors. *N. Engl. J. Med.*, **320**, 473.

FORREST, A.P.M., ROBERTS, M., CANT, E. & SHIVAS, A.A. (1976). Simple mastectomy and pectoral node biopsy. *Br. J. Surg.*, **63**, 569.

FRIEDMAN, M.A., DORR, F.A. & PERLOFF, M. (1986). Adjuvant therapy for breast cancer patients with negative lymph nodes. *NCI Monogr.*, **1**, 139.

JAKESZ, R., KOLB, REINER, G., SCHEMPER, M., RAINER, H. & DITTRICH, C. (1987). Adjuvant chemotherapy in node-negative breast cancer patients. In *Adjuvant Therapy of Cancer V*, Salmon, S.E. (ed.) p. 223. Grune & Stratton: Orlando, FL.

KAPLAN, E.L. & MEIER, P. (1958). Nonparametric estimation from incomplete observations. *J. Am. Stat. Assoc.*, **53**, 457.

KOYAMA, H., WADA, T., TAKAHASHI, Y. & 7 others (1980). Surgical adjuvant chemotherapy with mitomycin C and cylophosphamide in Japanese patients with breast cancer. *Cancer*, **46**, 2373.

LUDWIG BREAST CANCER STUDY GROUP (1989). Prolonged disease-free survival after one course of perioperative adjuvant chemotherapy for node-negative breast cancer. *N. Engl. J. Med.*, **320**, 491.

MANSOUR, E.G., GRAY, R., SHATILA, A.H. & 6 others (1989). Efficacy of adjuvant chemotherapy in high-risk node-negative breast cancer: an intergroup study. *N. Engl. J. Med.*, **320**, 485.

McGUIRE, W.L. & DE LA GARZA, M. (1973). Improved sensitivity in the measurement of estrogen receptor in human breast cancer. *J. Clin. Endocrinol. Metab.*, **37**, 986.

MORRISON, J.M., HOWELL, A., GRIEVE, R.J. & 4 others (1984). West Midlands Oncology Association trials of adjuvant chemotherapy for operable breast cancer. In *Adjuvant Therapy of Cancer IV*, Jones, S.E. & Salmon, S.E. (eds) p. 25 Grume & Stratton: New York.

MORRISON, J.M., HOWELL, A., GRIEVE, R.J., MONYPENNY, I.J., KELLY, K.A. & WATERHOUSE, J.A. (1987). West Midlands Oncology Association trials of adjuvant chemotherapy for operable breast cancer. In *Adjuvant Therapy of Cancer V*, Salmon, S.E. (ed.) p. 311. Grune & Stratton: Orlando, FL.

MORRISON, J.M., HOWELL, A., GRIEVE, R.J., MONEYPENNY, I.J., MINAWA, A. & WATERHOUSE, J.A. (1981). The West Midlands Oncology Association trials of adjuvant chemotherapy for operable breast cancer. In *Adjuvant Therapy of Cancer III*, Salmon, S.E. & Jones, S.E. (eds) p. 403. Grune & Stratton: New York.

NISSEN-MEYER, R., HØST, H., MÅNSSON, B. & NORIN, T. (1986). Treatment of node-negative breast cancer patients with short course of chemotherapy immediately after surgery. *NCI Monogr.*, **1**, 125.

PETO, R., PIKE, M.C., ARMITAGE, P. & 7 others (1977). Design and analysis of randomized clinical trials requiring prolonged observation of each patient: part II, analysis and examples. *Br. J. Cancer*, **35**, 1.

SEMIGLAZOV, V.F., BAVLI, J.L., MOISEYENKO, V.M. & 9 others (1986). Clinical trials on adjuvant chemotherapy for breast cancer. *Cancer*, **57**, 1957.

SENN, H.J., JUNGI, W.F., AMGWERD, R. & 7 others (1986). Swiss adjuvant trial (OSAKO 06/74) with chlorambucil, methotrexate, and 5-flourouracil plus BCG in node-negative breast cancer patients: Nine year results. *NCI Monogr.*, **1**, 129.

WILLIAMS, C.J., BUCHANAN, R.B., HALL, V. & TAYLOR, I. (1987). Adjuvant chemotherapy for T1-2, N0, M0 estrogen receptor negative breast cancer: preliminary results of a randomised trial. In *Adjuvant Therapy of Cancer V*, Salmon, S.E. (ed.) p. 233. Grune & Stratton: Orlando, FL.


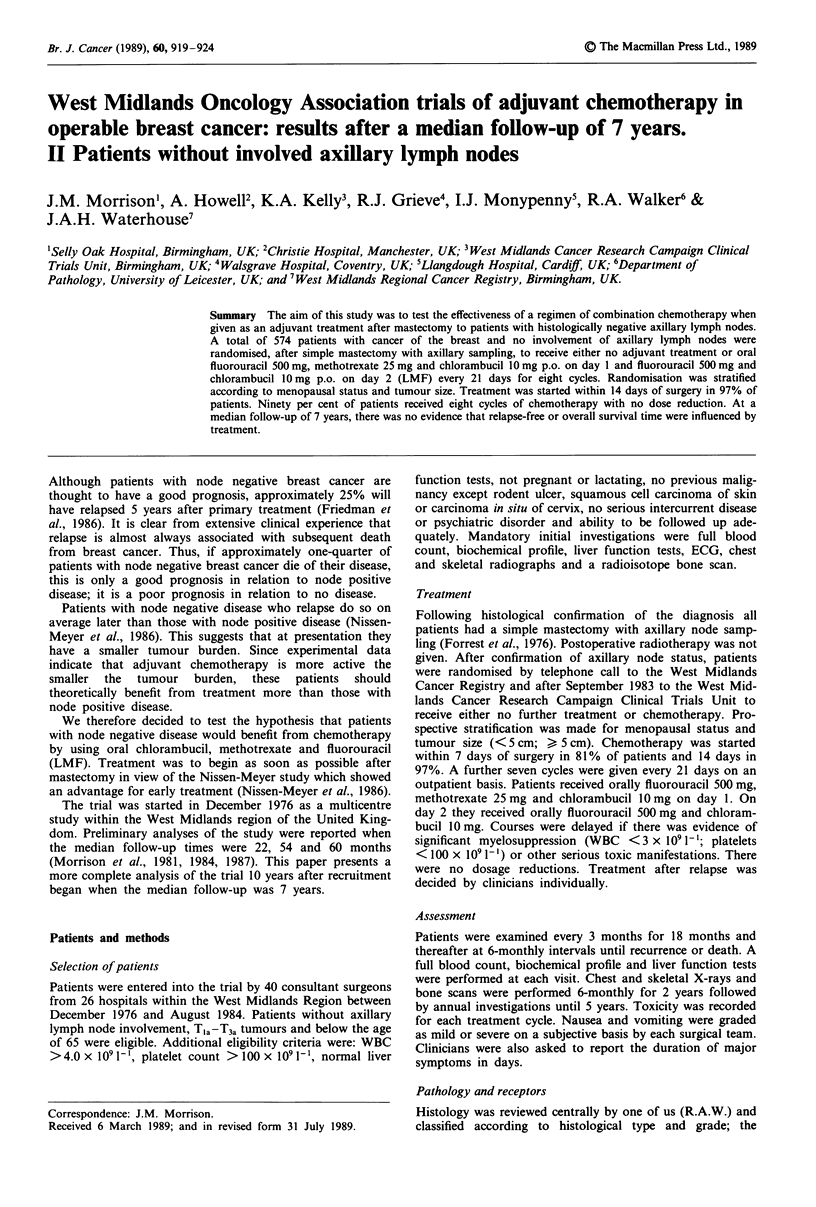

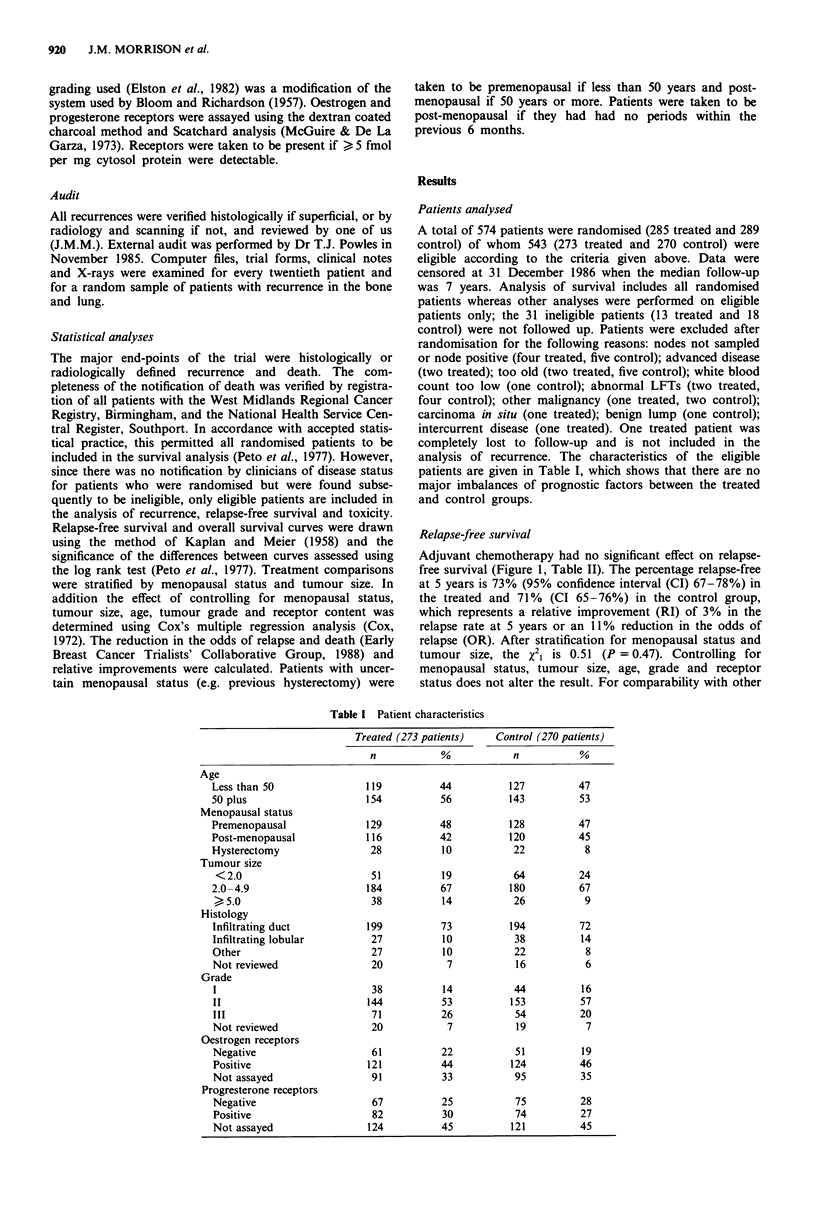

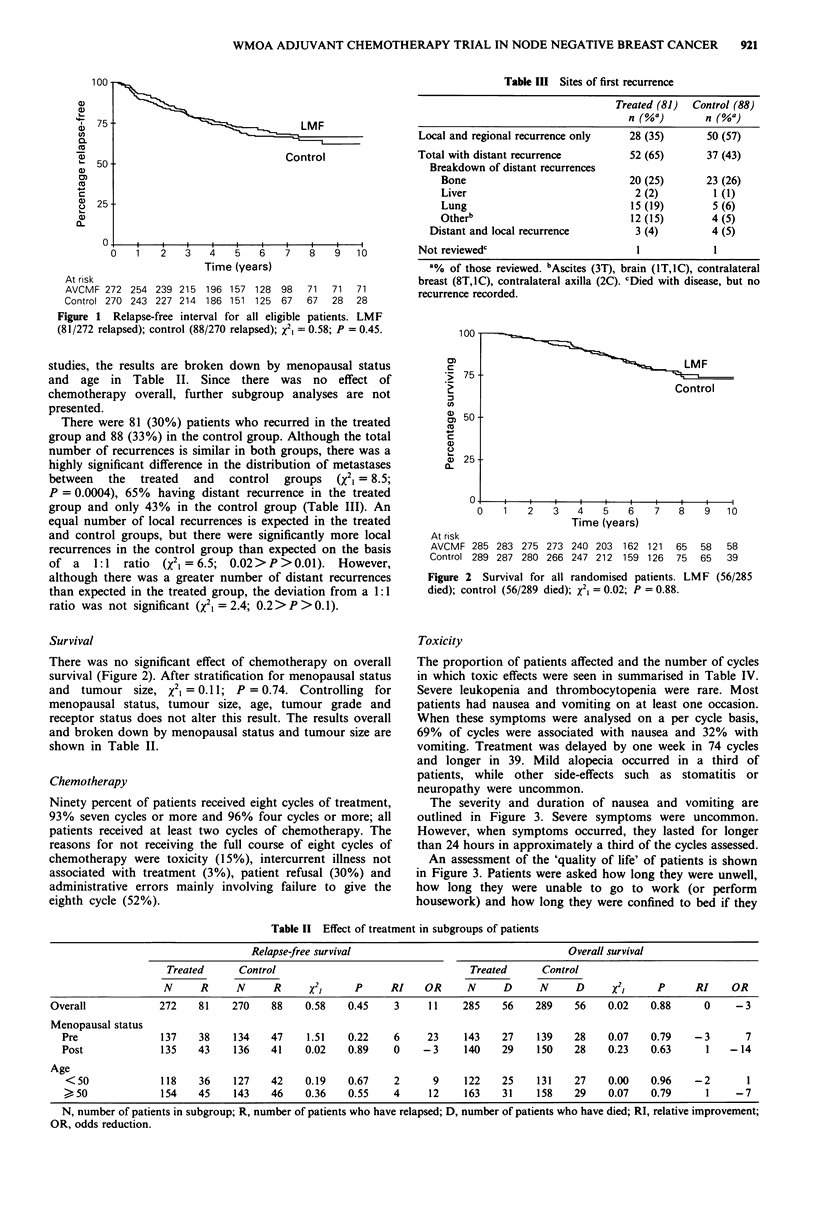

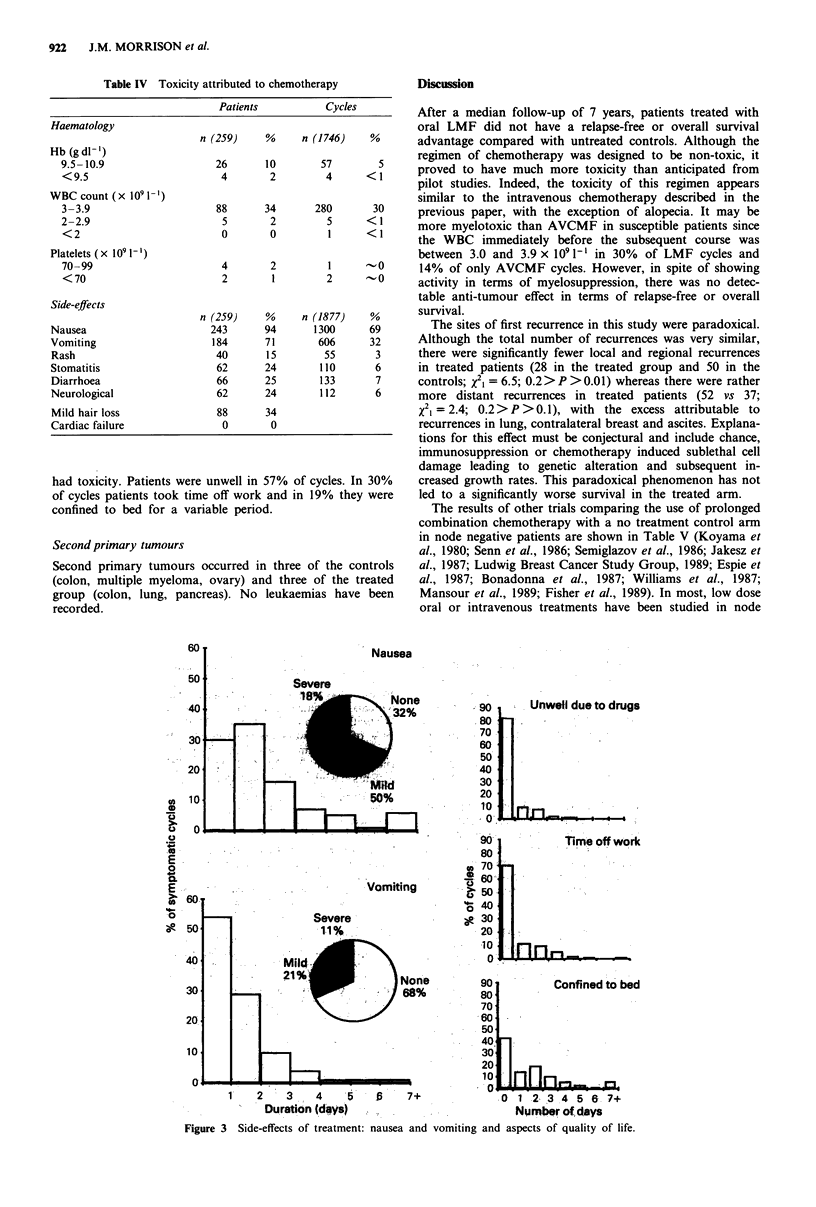

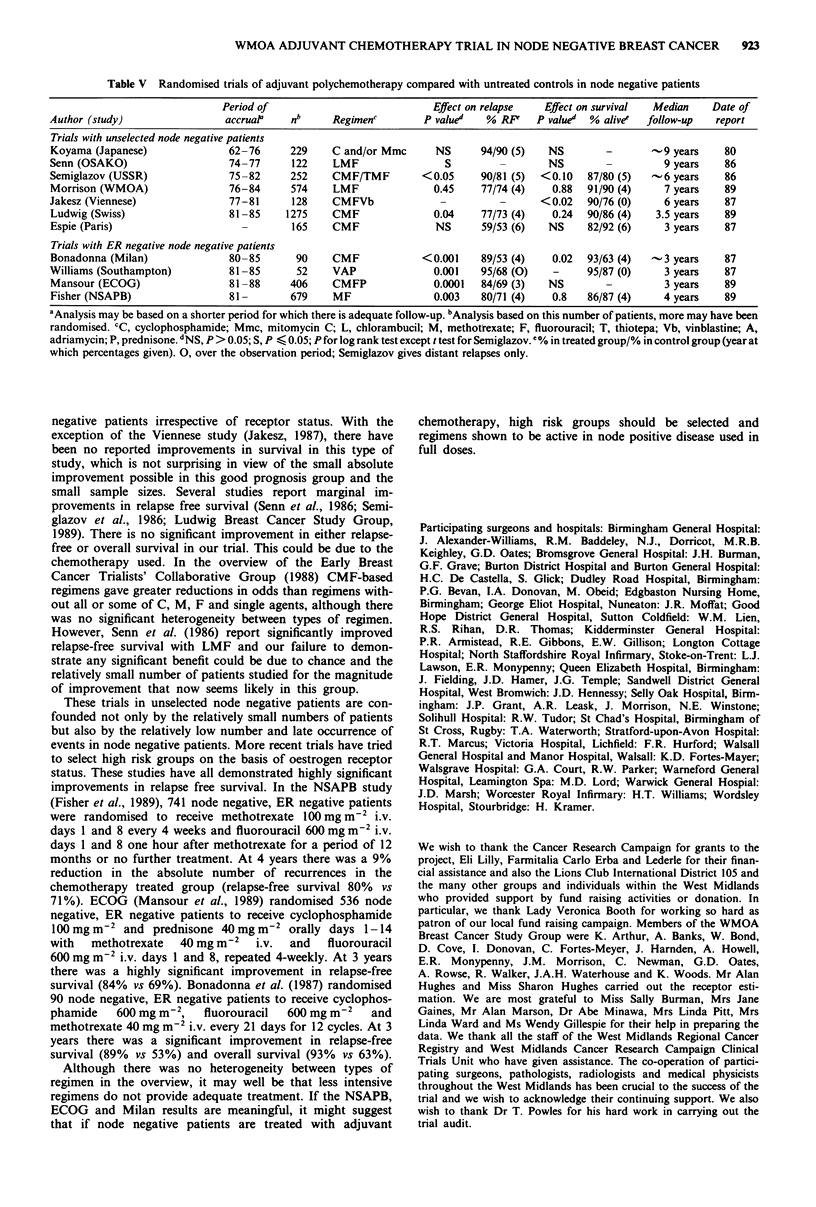

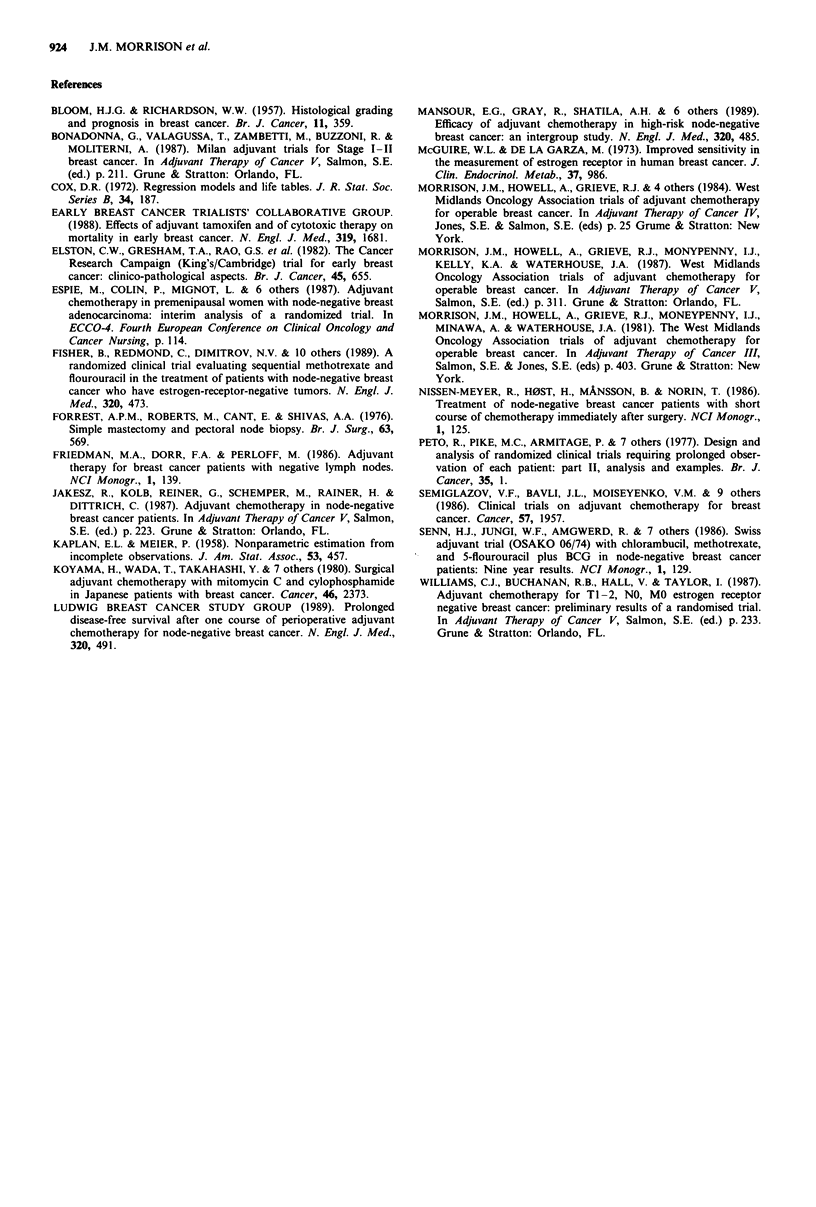


## References

[OCR_00909] BLOOM H. J., RICHARDSON W. W. (1957). Histological grading and prognosis in breast cancer; a study of 1409 cases of which 359 have been followed for 15 years.. Br J Cancer.

[OCR_00928] Elston C. W., Gresham G. A., Rao G. S., Zebro T., Haybittle J. L., Houghton J., Kearney G. (1982). The cancer research campaign (King's/Cambridge trial for early breast cancer: clinico-pathological aspects.. Br J Cancer.

[OCR_00940] Fisher B., Redmond C., Dimitrov N. V., Bowman D., Legault-Poisson S., Wickerham D. L., Wolmark N., Fisher E. R., Margolese R., Sutherland C. (1989). A randomized clinical trial evaluating sequential methotrexate and fluorouracil in the treatment of patients with node-negative breast cancer who have estrogen-receptor-negative tumors.. N Engl J Med.

[OCR_00947] Forrest A. P., Roberts M. M., Cant E., Shivas A. (1976). Simple mastectomy and pectoral node biopsy.. Br J Surg.

[OCR_00952] Friedman M. A., Dorr F. A., Perloff M. (1986). Adjuvant therapy for breast cancer patients with negative lymph nodes.. NCI Monogr.

[OCR_00967] Koyama H., Wada T., Takahashi Y., Nishizawa Y., Iwanaga T., Aoki Y., Terasawa T., Kosaki G., Kajita A., Wada A. (1980). Surgical adjuvant chemotherapy with mitomycin C and cyclophosphamide in Japanese patients with breast cancer.. Cancer.

[OCR_00978] Mansour E. G., Gray R., Shatila A. H., Osborne C. K., Tormey D. C., Gilchrist K. W., Cooper M. R., Falkson G. (1989). Efficacy of adjuvant chemotherapy in high-risk node-negative breast cancer. An intergroup study.. N Engl J Med.

[OCR_00982] McGuire W. L., DeLaGarza M. (1973). Improved sensitivity in the measurement of estrogen receptor in human breast cancer.. J Clin Endocrinol Metab.

[OCR_01009] Nissen-Meyer R., Høst H., Kjellgren K., Månsson B., Norin T. (1986). Treatment of node-negative breast cancer patients with short course of chemotherapy immediately after surgery.. NCI Monogr.

[OCR_01021] Semiglazov V. F., Bavli J. L., Moiseyenko V. M., Rzhankov S. V., Migmanova N. S., Popova R. T., Seleznyov I. K., Kremen B. V., Kostetskaya T. V., Barash N. J. (1986). Clinical trials on adjuvant chemotherapy for breast cancer.. Cancer.

[OCR_01026] Senn H. J., Jungi W. F., Amgwerd R., Hochuli E., Ammann J., Engelhart G., Heinz C., Wick A., Enderlin F., Creux G. (1986). Swiss adjuvant trial (OSAKO 06/74) with chlorambucil, methotrexate, and 5-fluorouracil plus BCG in node-negative breast cancer patients: nine-year results.. NCI Monogr.

